# Absorption, dispersion, and emission characteristics of novel Chitosan blends thin films tuned by UV-Ozone

**DOI:** 10.1038/s41598-026-40385-x

**Published:** 2026-03-21

**Authors:** E. A. Gaml, Hajer Abusnina, N. A. El-Ghamaz

**Affiliations:** 1 Department of Physics, Faculty of Science, Damietta University, Damietta, 34517 Egypt; 2 High and Intermediate Institute of Agricultural Technology-Gheran, Tripoli, Libya

**Keywords:** Chitosan, Blend, Optical, UV irradiation, Materials science, Optics and photonics, Physics

## Abstract

In current study, two novel polymers derived from original Chitosan is synthetized. Polymeric blends from Chitosan and the new polymers are used to fabricate spin coated thin films. The effect of UV irradiation on the optical properties of pristine Chitosan and two novel Chitosan derived polymers blends in form of thin film structure are studied. The optical parameters of the blends thin films such as the refractive index (n), extinction coefficient (k) and dielectric constants are determined by spectrophotometric technique in the spectral range 200–2500 nm. The energy gap values (E_g_), dielectric constants at infinity (ε_∞_), lattice permittivity (ε_L_) and dispersion parameters are calculated. E_g_ is found to be 5.14 eV for Chitosan film and it is reduced to 4.25 and 4 eV for the two polymeric blends. ε_∞_ is calculated to be 2.96 for Chitosan film which is reduced to 2.88 upon UV irradiation. The influence of exposing thin films to UV light demonstrates interesting modifications in linear and nonlinear optical properties (third order non-linear susceptibility and the non-linear refractive index) of Chitosan and its derivatives. The emission characteristics of the blends are also investigated. The fluorescence intensity of the polymeric films is decreased upon UV exposure. The obtained results propose that Chitosan and its derivatives are possible candidates for opto-electronic applications such as optical switching, optical limiters and OLED.

## Introduction

Chitosan is a natural biopolymer derived from Chitin^1,2^. It exhibits interesting optical properties due to its molecular structure, which makes it suitable for various applications, especially in biomedical and optical devices^1,3–12^. It is a potential candidate material for electro optical applications^13^. Chitosan and its derivatives have attracted considerable interest due to their antimicrobial and antifungal activity^14,15^. Due to its excellent properties especially in absorption, Chitosan can be used in a wide range of applications such as food processing, water treatment and sensors^16^. Opto- electronic applications on Chitosan are emerging. For instance, the development of sensors to detect metal ions and biological structures based on resonant excitation of surface free-electrons oscillations^17^. Recent studies revealed that measuring and understanding of the optical properties of materials are important^18,19^. In order to enhance electrical, optical, and thermal properties of Chitosan; metal oxides are extensively used with it as dopants^5–12^. Such Chitosan composites showed interesting optical and thermal properties which suggests that these blends can be used in opto-electronic applications. Other polymers blends showed improved electrical and optical properties by doping or by radiation^20–25^. Gami et al.^26^ have investigated the Nanocomposites of Chitosan (Cs)/polyacrylamide (PAM) with synthesized selenium nanoparticles (SeNPs) the UV/visible spectra showed an improvement in the optical properties of the polymeric samples. They found that all the films can be tailored in the optical and optoelectronic applications like as antireflection coating for solar cell and high refractive-index lens. In 2018^27^ Roshidi et al. have studied the structural and optical properties of Chitosan–poly(amidoamine) thin film have been prepared using a spin coating technique, The UV–vis absorption of Cs–PAMAM thin film was high optical band gap of 4.09 eV. Anas, et al.^28^ found that all the thin films showed emission peak at wavelength around 420 nm. From the UV–Vis spectra, bands at around 270 nm indicate newly opened band gaps which correspond to transitions from the highest occupied molecular orbital (HOMOs) to the s and p orbitals of the lowest unoccupied molecular orbits (LUMOs).

Optical characterization of thin films gives information about some important and therefore may be of permanent interest for several different applications^29^. Our previous studies on the effect of ultra violet irradiation on the optical properties of organic materials showed tuned optical parameters and modified morphology of the thin films^30–33^. Youn et al.^34^ reported that chitosan is strongly affected by UV light. They studied the bleaching effect of UV light on Chitosan polymer. Several studies have been reported on the effect of UV irradiation on Chitosan characteristics. Sionkowska et al.^35–38^ reported the influence of UV irradiation on different characteristics of Chitosan and blends containing Chitosan. They reported on the effect of UV light on mechanical and thermal properties of Chitosan and they found that it is strongly affected by it. They also studied the effect of UV light on surface properties and on biological activity. Also, modified Chitosan and Chitosan blends were studied under the influence of UV irradiation^38–42^. In our previous work^43^, thermal and dielectric properties of novel chitosan derived polymers were reported. The derived polymers were referred to as CHI-ph and CHI-OHph. These polymers showed good thermal stability and promising dielectric properties in the studied range of temperature. In current work, we aim to prepare blends of chitosan with the new polymers and to study the influence of UV irradiation on optical characteristics of Chitosan polymer and these blends in form of thin films.

## Experimental techniques

### Materials

**Fig. 1:**
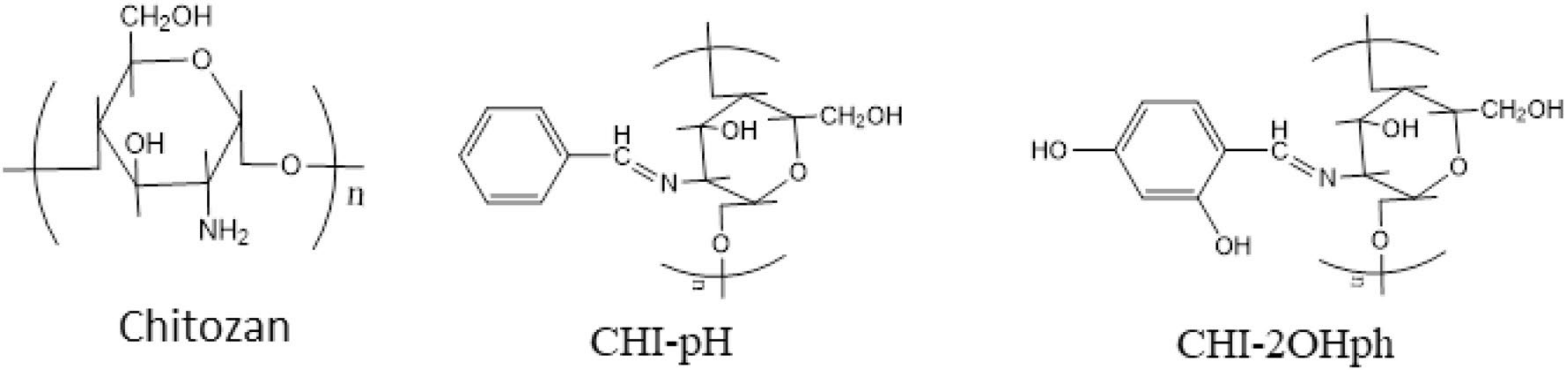
The molecular structural of Chitosan and its derivatives CHI-pH and CHI-2OHph.

In the present study Chitosan (**CHI**-**0**) was used as purchased. Two novel polymers were derived from chitosan as start material. The derived polymers are: E)-3-(benzylideneamino)-5-ethyl-6-(hydroxymethyl)-2- (methoxymethyl)-2,3,4,5,6-pentamethyltetrahydro-2H- pyran-4-ol (**CHI**-**pH**) and (E)-4-(((5-ethyl-4-hydroxy-6-(hydroxymethyl)-2-(methoxymethyl)-2,3,4,5,6-pentamethyltetrahydro-2H- pyran-3-ayl)imino)methyl)benzene-1,3-diol (**CHI**-**2OHph)**^43^**.** The polymers chemical structure is depicted in Fig. 1.

The derivatives CHI-pH and CHI-2OHph have been synthesized according to the method reported by Diab et. al.^44^. It found that CHI-pH and CHI-2OHph are insoluble in its pure form, so it’s worth to study these optical properties in the form of mixture of Chitosan with, CHI-pH and CHI-2OHph with ratio 2:1 respectively. **CHI-1** represents blend of chitosan and CHI-pH polymers. Also, **CHI-2** represents blend of chitosan and CHI-2OHph polymers.

### Samples preparation and experimental technique

The solution of pure CHI-0 is prepared by dissolving 0.1 g in 4 mL acetic acid solution and 2 ml distilled water and stirred for 12 h at room temperature. The polymers blends are prepared with ratio 2:1 of Chitosan to CHI-pH or CHI-2OHph. The thin films of CHI-0, CHI-1 and CHI-2 are prepared by spin coating technique onto quartz and glass substrates using VTC-50A Spin Coater at 1500 rpm for coating and 2500 rpm for drying for 30 s. The thicknesses of the thin films were measured experimentally using LEOI-44 Experimental Ellipsometer. Details of the measuring theory is illustrated elsewhere^45,46^. The thin films thickness is measured to be 300 nm.

FT-IR spectra are recorded by FT-IR (FTIR 430-JASCO, Japan) in the wavenumber range 4000–400 cm^−1^. The XRD measurements of the powder form performed of pure Chitosan and its derivatives using (Shimadzu XRD 6000) in the rang 4–80°, with step of 0.01 degrees/Sec. with Cu Kα radiation source.

The transmittance T (λ) and reflectance R(λ) of pure Chitosan thin films and chitosan blends are recorded by JASCO model V-570 UV- vis–NIR in the range 200–2500 nm. The accuracy in T (λ) and R(λ) measurement is 0.1% given by the instrument. Also, the errors of the optical constants are calculated to be 0.35, 2 and 2% for n, k and α, respectively^31^. The Photoluminescence spectra of thin films on glass substrate are recorded by Fluorimeter spectrophotometer (Model 6285, for Excitation and Emission spectra, 200–700 nm) with the excitation light with wavelength 217 nm. All measurements are performed in ambient room temperature.

### UV irradiation of thin films

The UV irradiation of the deposited CHI-0, CHI-1 and CHI-2 thin films was carried out using a highly intense UV-Ozone lamp. The UV- ozone lamp has wavelength around 254 nm (4.88 eV). The CHI-0, CHI-1 and CHI-2 thin films were exposed to UV-ozone irradiation for 20 min, to be compared with the as deposited thin films.

## Results and discussions

### Structural properties of Chitosan and its derivatives

The structural properties of polymers significantly influence their optical behavior. Additionally, understanding chemical composition modifications can provide insights into changes in optical constants and functionalities.

#### X-ray diffraction

**Fig. 2:**
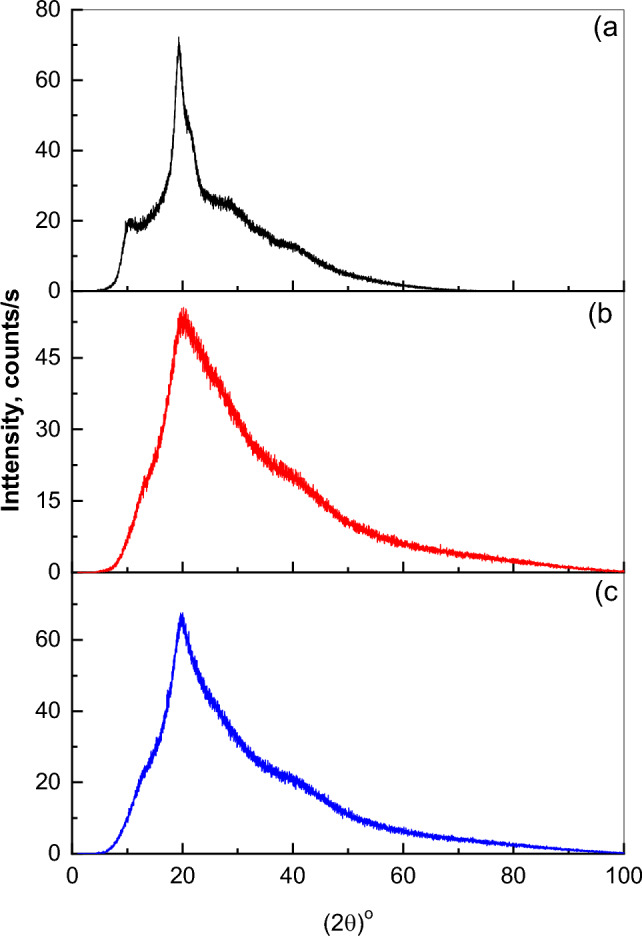
(**a**–**c**) XRD patterns of CHI-0, CHI-pH and CHI-2OHph.

The X-ray diffraction patterns (XRD) for pristine Chitosan (CHI-0) and its chemically modified versions CHI-pH and CHI-2OHph in the form of powder are shown in Fig. 2. The XRD patterns of the three polymers display broad peak at around 20° in addition to a low intense peak at 10° for chitosan. This shows that all the polymers demonstrate the standard behavior of amorphous Chitosan^47^. The chemical reaction introduced phenyl and hydroxyl groups in CHI-ph and CHI-OHph which results in more broadening of the peak at 20° and the vanishing of the peak at 10° indicating that more disorder occurred in the chitosan chain. Such broadening is a result of structural disorders due to the disrupt of the hydrogen bonding between chitosan chains^43^.

#### Fourier transforms infrared (FTIR) spectroscopy

**Fig. 3:**
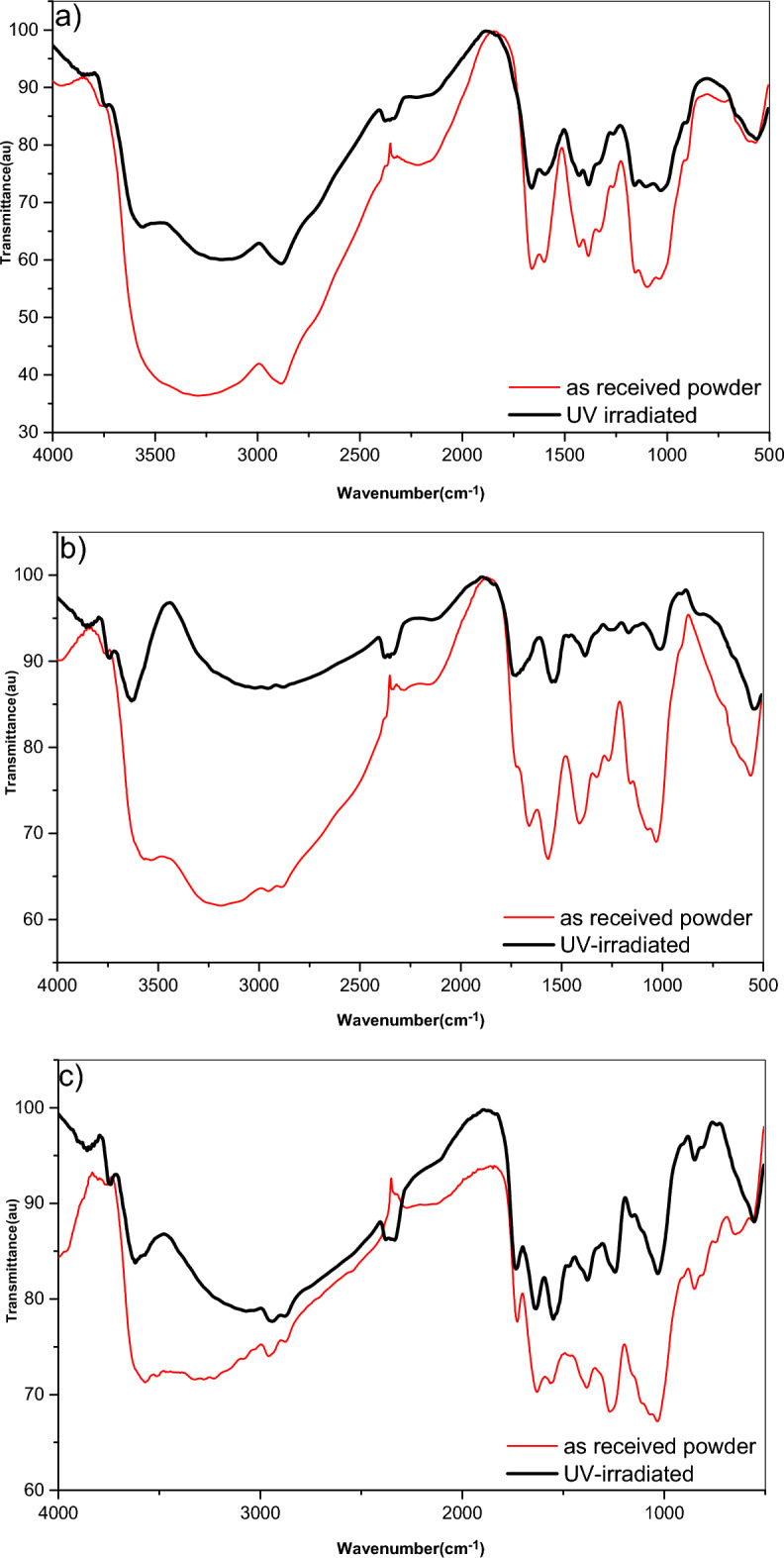
(**a**–**c**) FTIR spectra of as received and UV irradiated conditions for (**a**) CHI-0, (**b**) CHI-1 and (**c**) CHI-2 in powder form.

A comparison between the FTIR spectrum of as received powder of Chitosan and its derived polymers (CHI-pH and CHI-2OHph) and their UV irradiated powders are shown in Fig. 3a–c, respectively. Considering the spectra of as received and UV irradiated Chitosan (Fig. 1a), the spectra can be analyzed as follows. The broad band around 3200–3600 cm⁻^1^ is characteristic of O–H stretching vibrations, primarily from the hydroxyl groups in Chitosan.

The sharp peak around 1590 cm⁻^1^ and the peak around 1320 cm⁻^1^ are indication of N–H bending vibrations and C-N stretching vibrations, respectively, associated with the amide groups in Chitosan. The strong peak around 1650 cm⁻^1^ corresponds to C = O stretching vibrations, also it is related to the amide groups. The peak around 1020–1100 cm⁻^1^ is attributed to C–O–C stretching vibrations. The intensity of the O–H stretching band appears to be slightly reduced in the UV-irradiated sample, suggesting a possible decrease in hydroxyl groups which could be due to photo degradation process. For the spectra of the two derived polymers (Fig. 3b,c); the same absorption peaks appeared and changes due to UV irradiation still the main feature as for Chitosan in addition to the appearance of the absorption peaks around 3030–3100 cm⁻^1^ and 1600–1450 cm⁻^1^ which indicate the presence of a benzene ring.

## Optical characterization

### The spectral behavior of transmittance, T, and reflectance, R

**Fig. 4:**
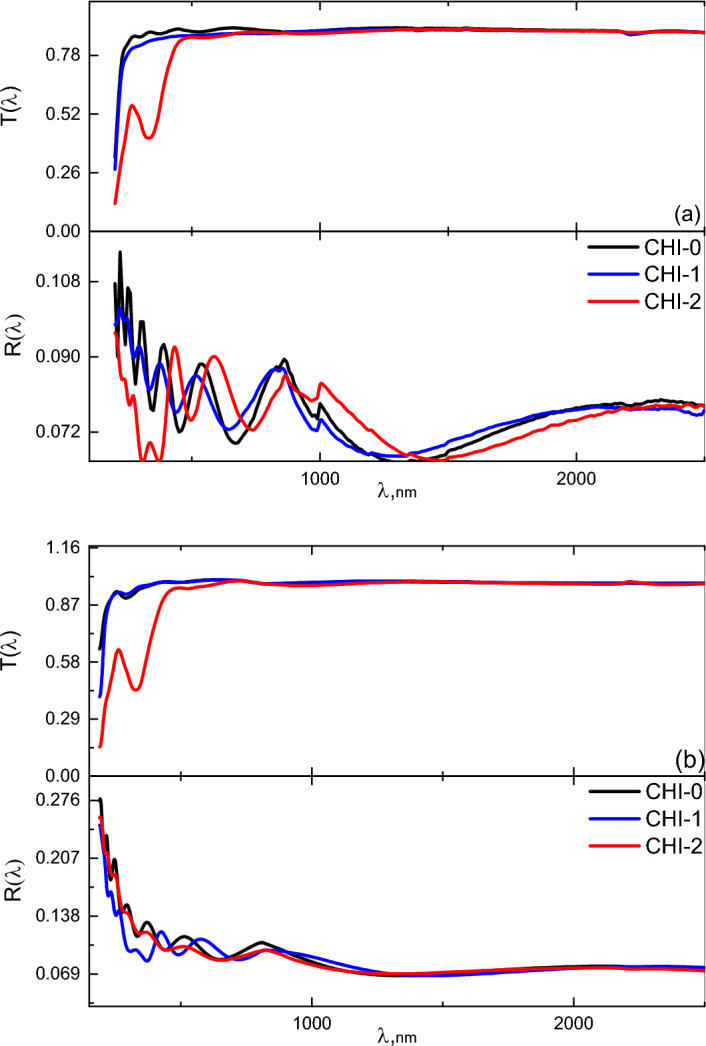
*T* and *R* spectra of CHI-0, CHI-1 and CHI-2 in (**a**) as prepared and (**b**) as irradiation condition.

Figure 4 illustrates the spectral distribution of transmittance (T) and reflectance (R) for Chitosan (CHI-0) and its blends (CHI-1 and CHI-2) in both as-prepared and UV-irradiated conditions, across the wavelength range of 200–2500 nm. Upon UV irradiation, a notable increase in transmittance is observed across the entire spectral range. Additionally, the reflectance spectra exhibit smoother profiles, suggesting a reduction in surface roughness. Furthermore, the shifts in the absorption bands within the 200–600 nm range indicate potential rearrangement of polymer molecules, leading to increased film compactness.

### Refractive index (n) and dispersion parameters

Refractive index (n) and absorption index (k) are fundamental parameters in the design and optimization of integrated optical devices. The complex refractive index, $\tilde{n}\,$, is given by the relation^48^:

1 $$\tilde{n}\, = n\, - \,ik$$

The refractive index, n, can be calculated from the absolute reflectance, R, of the material and its extinction coefficient, k, by using the following relation^48^:

2 $$n = \frac{(1 + R)}{{(1 - R)}} + \sqrt {\left( {\left( {\frac{4R}{{(1 - R)^{2} }}} \right) - k^{2} } \right)}$$

**Fig. 5:**
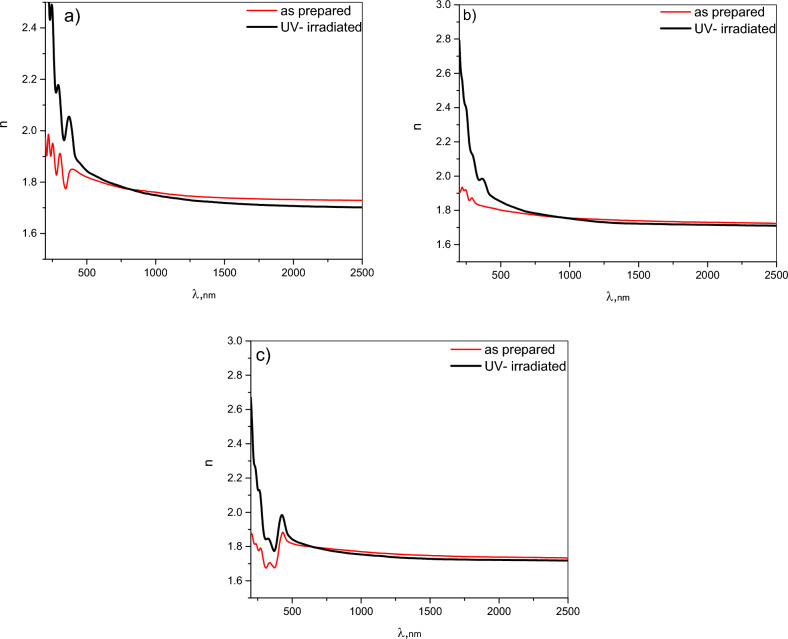
Spectral distribution of *n* for; (**a**) CHI-0, (**b**) CHI-1 and (**c**) CHI-2, thin films respectively.

The spectral behavior of the refractive index, n, for the as deposited CHI-0, CHI-1 and CHI-2 thin films and the effect of UV irradiation on it are presented in Fig. 5a–c, respectively.

The spectral behaviors of ***n*** for the three polymeric films are characterized by anomalous dispersion due to the multi-oscillator model^49^, caused by multiple electronic transition from bonding to anti bonding molecular orbitals in the wavelength range 200–600 nm. Also, a normal dispersion behavior of n appeared in wavelength range 600–2500 nm due to the absence of electronic transitions in this region of the incident photon energy (transparent region).

For all polymeric thin films; it noticed that n is more sensitive to UV irradiation in the anomalous dispersion region where ***n*** increased obviously. The increase in the refractive index may be attributed to cross-linking induced between polymers chains upon UV irradiation, leading to changes in the material’s density and consequently the refractive index. Furthermore, the exposure to UV radiation may induce changes in the material’s electronic properties, leading to a noticeable increase in the refractive index. In the normal dispersion region, Chitosan polymers experience only a slight decrease due to weaker interactions.

The UV-induced changes in the refractive index can be harnessed for various optical applications such as optical sensors for radiation and waveguide fabrication where irradiating specific regions of the thin film can create waveguides with tailored refractive index profiles.

**Fig. 6:**
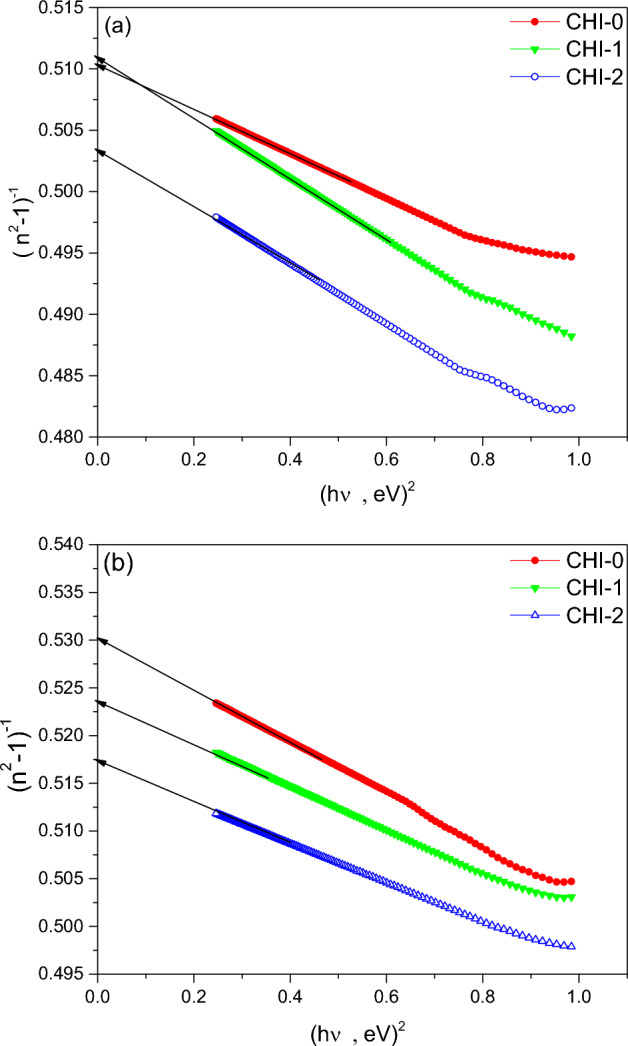
**(n**^*2*^*-1)*^*-1*^ versus *(hν)*^*2*^ spectra for CHI-0, CHI-1 and CHI-2 in (**a**) as prepared and (**b**) UV irradiated conditions.

The refractive index for CHI-0, CHI-1 and CHI-2 thin films gradually decreases in the normal dispersion region of the spectra. In this region, the refractive index is related to the incident photon energy (hν) by the relation^50,51^:

3 $$\frac{1}{{n^{2} - 1}} = \frac{{E_{o} }}{{E_{d} }} - \frac{1}{{E_{o} E_{d} }}(h\upsilon )^{2}$$

where E_o_ and E_d_ are the oscillator and the dispersion energies, respectively. Figure 6a,b shows the relationship between (n^2^-1)^-1^ and incident photon energy (hν) for thin films of Chitosan, CHI-1 and CHI-2 in both as-prepared and UV-irradiated conditions. The dielectric constant at high frequency (ε_∞_) can be determined by extrapolating the linear portion of the curve to the y-axis at hν = 0. This intersection point equals (ε_∞_-1)^-1^. The values of E_o_, E_d_, and ε_∞_ were calculated from the slope and intercept of the linear fit and are listed in Table 1.

**Table 1:** Dispersion parameters of three samples thin films.

Parameters	CHI-0		CHI-1		CHI-2	
	As prepared	UV- irradiated	As prepared	UV- irradiated	As prepared	UV- irradiated
E_o_ (eV)	5.27	4.44	4.6	4.7	4.53	5.02
E_d_ (eV)	10.33	8.37	8.98	9.1	9	9.7
$\varepsilon_{\infty }$	2.96	2.88	2.96	2.91	2.98	2.93
$\varepsilon_{L}$	3.02	2.96	3.04	2.97	3.06	2.99
N/m*(kg m^−3^)	7.8810^27^	2 × 10^28^	1.55 × 10^28^	1.78 × 10^28^	1.18 × 10^28^	8.91 × 10^28^

**Fig. 7:**
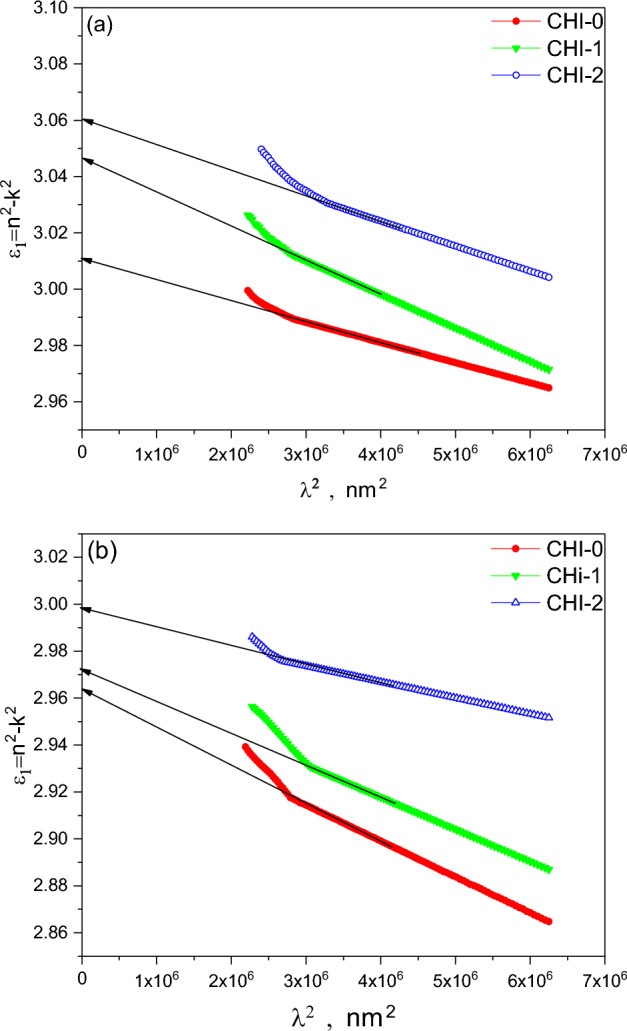
*n*^*2*^ versus λ^2^ spectra for CHI-0, CHI-1 and CHI-2 in (**a**) as prepared and (**b**) UV irradiated conditions.

Other optical parameters such as the lattice dielectric constant, ε_L_, and the ratio of carrier concentration to the effective mass, N/m*, can be calculated according to the following equation^52^:

4 $$\varepsilon _{1} = n^{2} - k^{2} = \varepsilon _{L} - \left( {\frac{{e^{2} }}{{4\pi ^{2} c^{2} \varepsilon _{o} }}} \right)\left( {\frac{N}{{m^{*} }}} \right)\lambda ^{2}$$

where **e** is the electron charge and **c** is the speed of the light. Figure 7a,b shows the relationship between $\varepsilon_{1} = n^{2} - k^{2}$ and λ^2^ for thin films of Chitosan and blends; CHI-1 and CHI-2 in both as-prepared and UV-irradiated conditions. The linear fit of the experimental data yields the values of N/m* and ε_L_, which are listed in Table 1. Upon UV irradiation, both ε_∞_ and ε_L_ are decreased slightly for all polymeric blends. This reduction may be attributed to structural changes induced by the UV radiation. Interestingly, N/m* increased slightly for Chitosan and CHI-1blend, while it decreased for CHI-2. The decrease in ε_∞_ suggests a reduction in the polymer’s ability to polarize electronically in response to the applied electric field. This could be due to changes in the electronic structure and the induced cross-linking between polymer chains upon UV irradiation. The decrease in ε_L_ indicates a change in the vibrational properties of the polymeric matrix. This could be due to changes in bond strength, intermolecular forces, or mass density caused by UV irradiation.

### Absorption index (k)

**Fig. 8:**
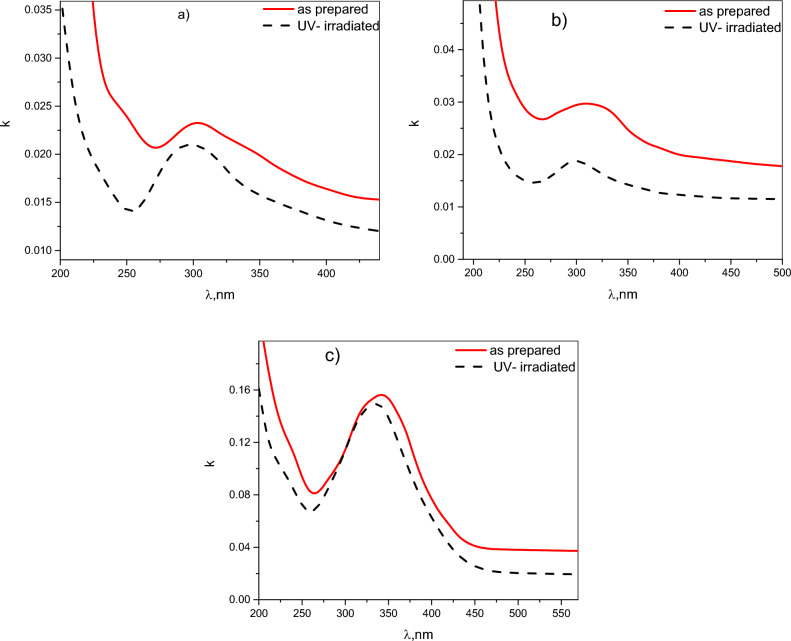
The spectral behaviors of the extinction coefficient, *k* for; CHI-0, CHI-1 and CHI-2, thin films respectively.

The extinction coefficient or absorption index (k) is the imaginary part of the complex refractive index. It measures how strongly a material absorbs light at a specific wavelength. Figure 8a–c illustrates the spectral distribution of k for thin films of pristine Chitosan (CHI-0) and its blends; CHI-1 and CHI-2, respectively, in the as-prepared and UV-irradiated conditions. All polymeric blends exhibit a primary absorption peak, with the peak position varying among the different polymers. Upon UV irradiation; the absorption index of all polymers decreases across the entire wavelength range. Additionally, the peak in the UV-irradiated samples shifts slightly towards longer wavelengths. These changes in the absorption spectra suggest that UV irradiation induces alterations in the electronic structure of the chitosan and its derivatives. The reduced absorption could be attributed to the annealing of defects or changes in the electronic structure. By understanding these mechanisms, the optical properties of Chitosan-based materials can be optimized by tailoring the UV irradiation process.

### Dielectric constants

The dielectric function links solid’s electronic transitions to its structure, enabling the extraction of valuable band structure information from its dielectric spectrum. The real component (ε_r_) and the imaginary component (ε_i_) of the complex dielectric constant (ε*) are interconnected by the following equations^7^:

5 $$\varepsilon^{ * } \, = \varepsilon_{r} \, - \,i\,\,\varepsilon_{i}$$

where

6 $$\varepsilon_{r}^{{}} = n^{2} - k^{2}$$

and

7 $$\varepsilon_{i} \, = 2nk$$

**Fig. 9:**
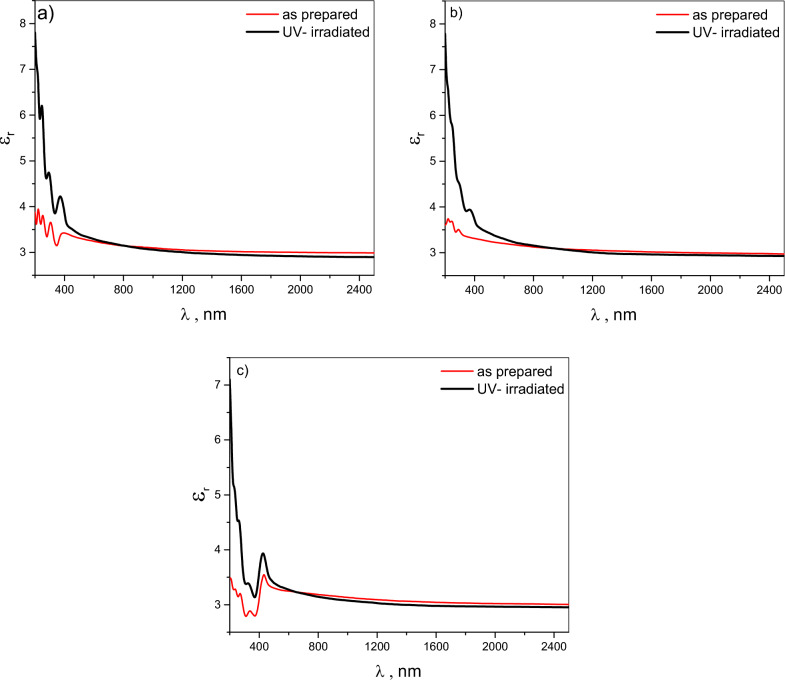
(**a**–**c**): Spectral behavior of real dielectric constant ɛr for CHI-0, CHI-1 and CHI-2 thin films respectively.

**Fig. 10:**
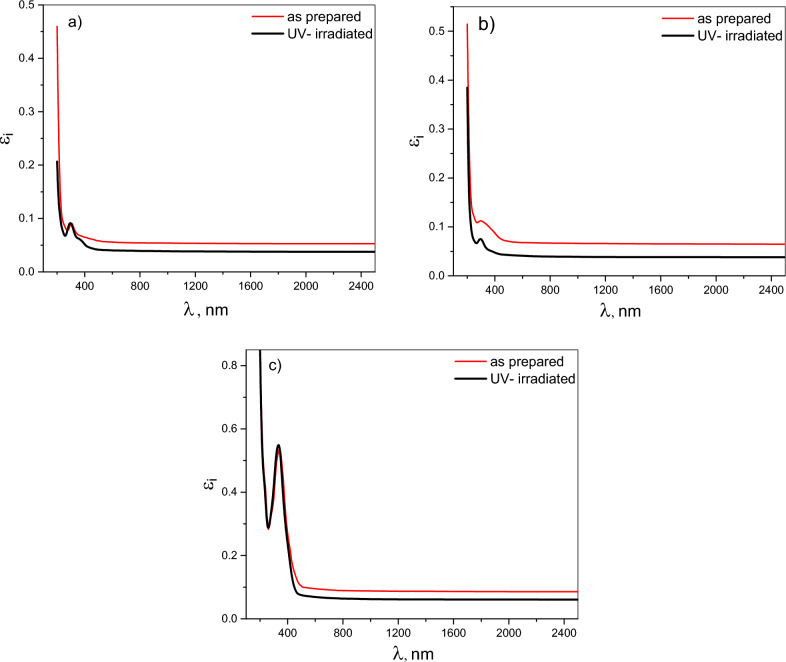
(**a**–**c**) Spectral behavior of imaginary dielectric constant ɛi for CHI-0, CHI-1 and CHI-2 thin films respectively.

The spectral characteristics of the real (ε_r_) and imaginary (ε_i_) components of the dielectric constant are modulated by variations in both the refractive index (n) and extinction coefficient (k). Figures 9 and 10 depict the influence of UV irradiation on the spectral behavior of ε_r_ and ε_i_ for pristine Chitosan and its blends (CHI-1 and CHI-2) thin films. In general, the ε_r_ values exhibit a higher magnitude compared to the ε_i_ values for chitosan and its blends. The spectral distribution of ε_r_ and ε_i_ signifies diverse interactions between incident photons and the investigated films. The peaks observed within the wavelength range of 200–450 nm are attributed to electronic transitions from bonding to anti-bonding molecular orbitals, induced by photon absorption. Modifications to the chitosan molecule changed the peak position. Normal dispersion is exhibited by all samples at wavelengths exceeding 2500 nm. This indicates a more stable interaction with light, where the dielectric properties become less sensitive to changes in wavelength, suggesting a consistent material response over a broader range of conditions.

Exposure to UV radiation modulates both ε_r_ and ε_i_ in disparate manners. ε_r_ for the three polymeric thin films under investigation demonstrates a substantial increase in its value within the wavelength range of 200–600 nm, whereas a subtle decrease in its value is observed due to stable interaction with incident light in the normal dispersion region of the spectra. The increase in ε_r_ may be ascribed to an increase in the refractive index, as well as modifications in the electronic structure of Chitosan and its two derivatives under investigation. The significant increase in ε_r_ across the 200–600 nm wavelength range indicates that enhanced light-matter interactions following UV exposure and recommend these materials for potential applications in optoelectronic devices, such as waveguides and nonlinear optics. UV-irradiation exhibits a limited effect on the spectral behavior of ε_i_, with only minor alterations in absorption features, reflecting modifications to the electronic structure induced by UV- irradiation. Furthermore, the limited impact on ε_i_ suggests that these materials may maintain their performance characteristics under operational conditions, making them suitable candidates for further exploration in various optoelectronic applications.

### Absorption characteristics and determination of Energy gap:

The absorption coefficient, α, of the thin-film material can be determined from the measured absorption index (k) spectrum using the following equation^52^:

8 $$\alpha \, = \,\frac{4\,\pi \,k}{\lambda }\,\,$$

**Fig. 11:**
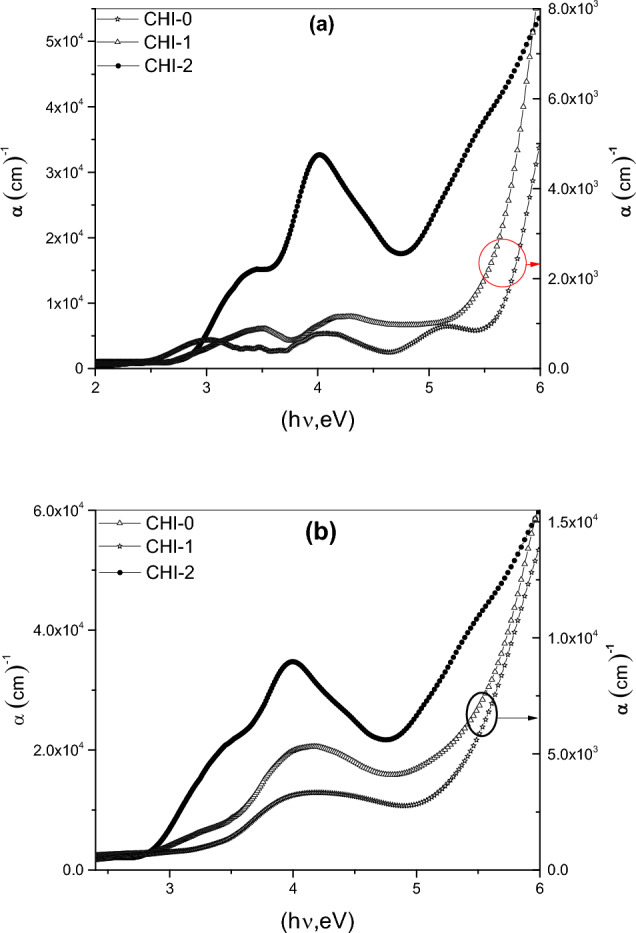
Absorption coefficient *α* for CHI-0, CHI-1 and CHI-2 in (**a**) pristine and (**b**) UV- irradiated condition.

Figure 11a and b illustrate the calculated α values for the three polymeric thin films in their original and UV-irradiated states as a function of incident photon energy (hν). The absorption coefficient exhibits multiple peaks, which correspond to electronic transitions between bonding and anti-bonding molecular orbitals. These transitions can be interpreted using the multi-oscillator model^53^. Table 2 summarizes the peak positions and assignments of the electronic transitions for chitosan and its blends.

**Table 2:** Peak position of absorption bands for CHI-0, CHI-1 and CHI-2.

Peak position					
CHI-0		CHI-1		CHI-2	
As prepared	UV- irradiated	As prepared	UV- irradiated	As prepared	UV- irradiated
5.14 (π—π*)	4.13(π-π* strong)	4.25 (π-π*)	4.4 (π- π*)	4 (π-π*)	3.98 (π- π* strong)
4.08 (π- π*)	–	–	–	–	–
3 (π- π* weak)	3.2 (π-π* shoulder)	3.49 (π-π* shoulder)	3(π- π* shoulder)	3.51(π- π* shoulder)	3.47(π-π* shoulder)

Energy gap (E_g_) of an organic molecular material is an important parameter in constructing energy band diagram and should be taken into consideration in designing optoelectronic devices. The spectral distribution of α near the absorption edge can be attributed to the electronic transitions between higher occupied molecular orbitals (HOMO) and lower unoccupied molecular orbitals (LUMO) of the molecule. The value of E_g_ and the types of electronic transition are useful in determine the suitable application and they can be estimated according to the following relation^54^:

9 $$\alpha \,h\,\nu = B(h\nu - E_{g} )^{r} ,$$

where *B* is a constant related to electrical conductivity and energy level separation, The value of *r* characterizes the transition process where r = ½ and 3/2 for direct allowed and forbidden transitions, respectively, and r takes the value of 2 and 3 for indirect allowed and forbidden transitions, respectively . Differentiating Eq. (9) with respect to hν and dividing Eq. (9) by the derivative yields the following relation^55^:

10 $$\left[ {{\raise0.7ex\hbox{${\alpha \,h\,\nu }$} \!\mathord{\left/ {\vphantom {{\alpha \,h\,\nu } {\left( {d\,\left( {\alpha \,h\,\nu } \right)/d\,\left( {\,h\,\nu } \right)} \right)}}}\right.\kern-0pt} \!\lower0.7ex\hbox{${\left( {d\,\left( {\alpha \,h\,\nu } \right)/d\,\left( {\,h\,\nu } \right)} \right)}$}}} \right] = \frac{1}{m}(h\nu - E_{g} ).\,\,$$

**Fig. 12:**
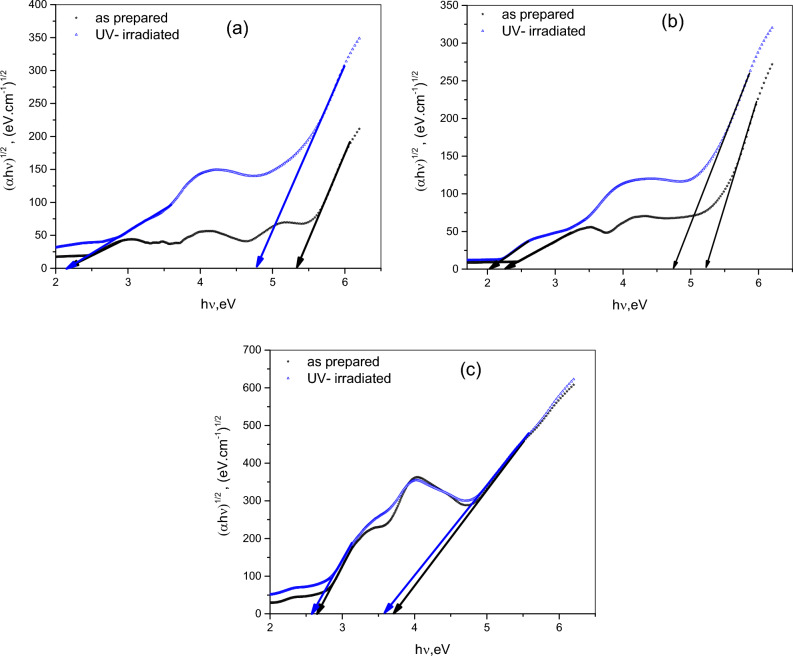
Dependence of (αhν)^1/2^ on hν for pristine and UV—irradiated for pristine and UV irradiated of (**a**) CHIi-0, (**b**) CHI-1 and (**c**) CHI-2 thin films.

A plot of $\,\alpha \,h\,\nu /\left( {d\,\left( {\alpha \,h\,\nu } \right)/d\,\left( {\,h\,\nu } \right)} \right)$ versus $h\nu$ is a straight line in the spectral range near the absorption edge with slope of 1/r of about 0.5 and so the best expected type of electronic transition is indirect allowed. The values of the optical band gap (E_g_) and onset gap (E_onset_) energies for CHI-0, CHI-1 and CHI-2 are determined by extrapolating the linear portion of the absorption edge to the x-axis, as shown in Figs. 10c, 12a,b respectively. These values are summarized in Table 3 for both pristine and UV-irradiated samples. It is observed that UV irradiation led to a decrease in the energy gap of the polymers. This reduction in E_g_ is likely due to structural changes in the polymer matrix and modifications to the electronic structure caused by UV exposure. Additionally, the Chitosan derivatives exhibited lower energy gaps compared to pure Chitosan, which can be attributed to the influence of the coupled functional groups. The role of function group is to facilitate the charge carrier movements through the polymer. In other words, adding functional groups to Chitosan made it easier for the material to absorb and release energy, which can be useful for various optoelectronic devices such as organic solar cells, organic photodetector and organic light-emitting diodes (OLEDs). Different polymeric blends were reported to have enhanced optical properties after being irradiated^56–58^.

**Table 3:** Onset, optical energy gap and Urbach tail for pristine and UV irradiation of CHI-0, CHI-1 and CHI-2 thin films.

Parameters	CHI-0		CHI-1		CHI-2	
	As prepared	UV- irradiated	As prepared	UV- irradiated	As prepared	UV- irradiated
E_g,_ (eV)	5.36	4.55	5.24	4.74	3.71	3.58
E_onset_	2.15	2.15	2.26	2.03	2.64	2.56
E_u,_ (eV)	0.31	0.96	0.35	0.72	0.20	0.39

**Fig. 13:**
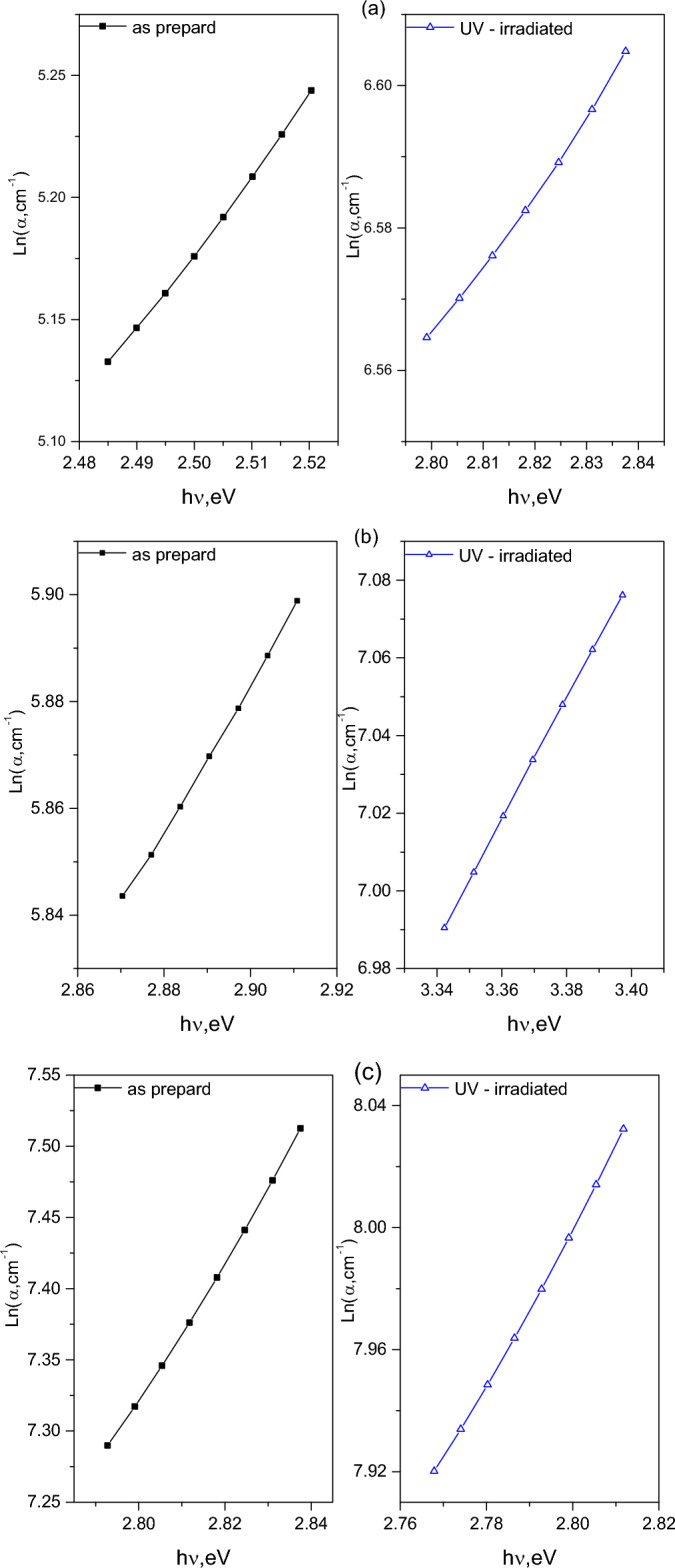
Plot of Ln α versus incident photon energy, hν, for pristine and UV- irradiated of (**a**) CHI-0, (**b**) CHI-1 and (**c**) CHI-2 thin films.

Amorphous semiconductors and insulators, like polymers, often exhibit localized states within their bandgaps. These localized states can be characterized using the Urbach relation^59^:

11 $$\alpha \, = \alpha_{o} \,\exp \,\left( {\frac{h\nu }{{E_{u} }}} \right)$$

where α_o_ is a pre-exponential constant and E_u_ represents the width of localized states within the band gap. By analyzing the relationship between the absorption coefficient (ln α) and the incident photon energy for both pristine and UV-irradiated polymers samples (as depicted in Fig. 13), the values of Eu were determined and tabulated in Table 3. The results generally indicate that UV irradiation leads to an increase in E_u_, which aligns with the observed decrease in the band gap energy (E_g_) following UV exposure.

### Non-linear optical parameters

**Fig. 14:**
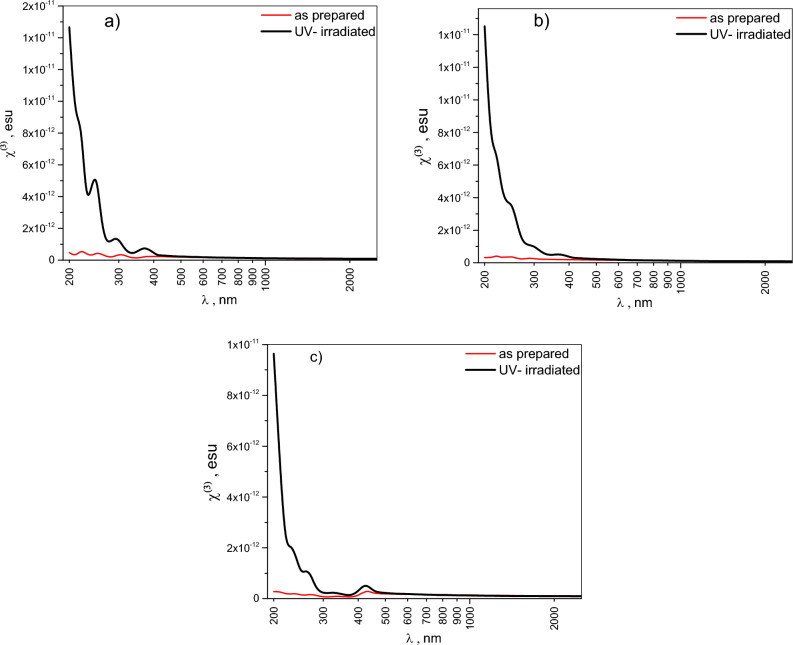
Plot third order nonlinear susceptibility χ^(3)^ versus incident photon energy, *λ*, for pristine and UV irradiated of (**a**) CHI-0, (**b**) CHI-1 and (**c**) CHI-2 thin films.

**Fig. 15:**
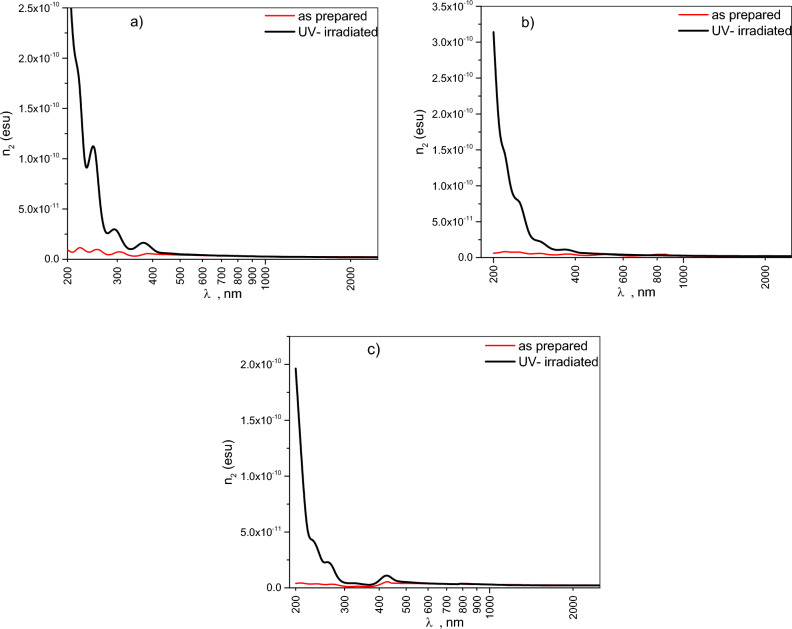
Plot the non-linear refractive index n_2_ versus incident photon energy, λ, for pristine and UV irradiated of (**a**) CHI-0, (**b**) CHI-1 and (**c**) CHI-2 thin films.

Nonlinear optical phenomena arise when materials interact with high-intensity electromagnetic fields^12^. The third-order nonlinear optical susceptibility χ^(3)^ and the nonlinear refractive index n_2_ are crucial parameters determining a material’s nonlinear optical response^60,61^. These parameters can be estimated using the following Tichy and Ticha relation^62^, which is derived from Miller’s rule^29^.

12 $$\chi^{(1)} = \,\,(n^{2} \, - \,1\,)\,/4\pi$$

13 $$\chi^{(3)} \, = A\,[\,\chi^{(1)} ]^{4}$$

14 $$n{}_{2\,}\, = \,\frac{12\,\pi }{{n_{o} }}\,\,\chi^{(3)}$$

where, A = 1.7 × 10^–10^ esu, and $n{}_{o}\, = \,\sqrt {\varepsilon_{\infty } }$,

The obtained results highlight the impact of UV-irradiation on the nonlinear optical properties χ^(3)^ and *n*_2_​ for CHI-0, CHI-1 and CHI-2 thin films as shown in Figs. 14 and 15. The results indicate that in the wavelength range 200–500 nm, UV irradiation leads to a significant increase in both parameters, while there is almost no change in the 500–2500 nm range. The enhancement in nonlinear parameters **χ**^**(3)**^ and n_2_ upon UV-irradiation reflects the permanent effect of UV-irradiation on the structural properties of Chitosan and its derivatives, making the materials suitable for applications such as optical switching and limiting, due to enhanced control over light flow. Additionally, materials with enhanced third-order susceptibility are strong candidates for engaging in two-photon absorption mechanisms, such as second harmonic generation, as indicated by studies on organic and in organic materials^30,64,65^.

### Emission spectra of chitosan and its derivatives

**Fig. 16:**
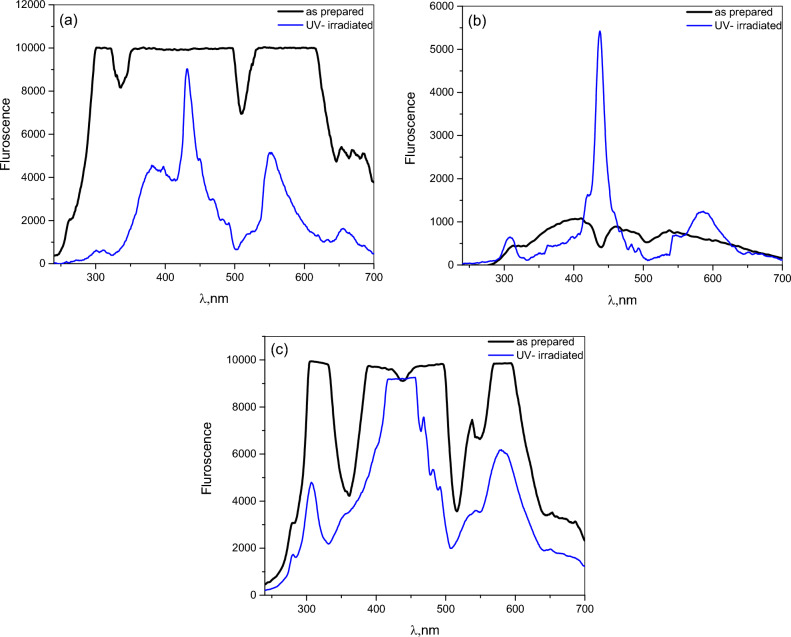
Emission spectra for (**a**) Chitosan (CHI-0) and its derivatives (**b**) CHI-1 and (**c**) CHI-2 thin films in as prepared and UV irradiated conditions.

The emission spectra of spin-coated Chitosan (CHI-0) and its derived polymers (CHI-1 and CHI-2) thin films were investigated under both pristine and UV-irradiated conditions. The spectra, depicted in Fig. 16, reveal broad emission bands extending from 250 to 700 nm, characterized by multiple peaks in the UV and visible regions. Upon exposure to UV radiation, the broad emission bands were resolved into more defined peaks, suggesting alterations in the electronic structure of the polymers due to photo degradation. Notably, while the emission intensity of CHI-0 and CHI-2 decreased, CHI-1 exhibited a significant enhancement in intensity at 437 nm.

**Fig. 17:**
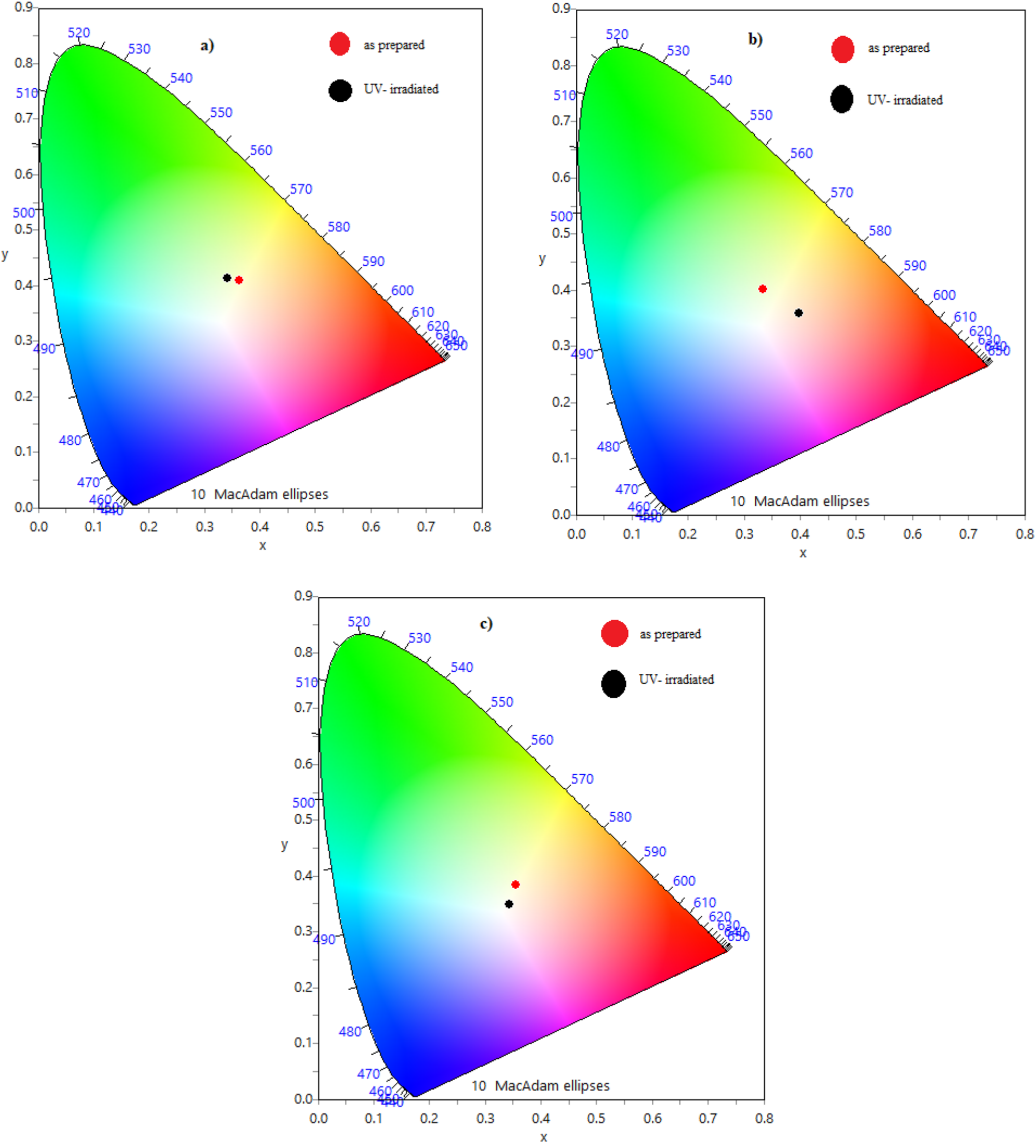
CIE diagram for: (**a**) CHI-0, (**b**) CHI-1 and (**c**) CHI-2 thin films in as prepared and UV irradiated conditions.

Colour coordinate calculations, performed using colour calculator software^66^, indicate that all samples emit white light, making them promising candidates for OLED applications in both indoor and outdoor environments. Figure 17 presents the CIE diagram for the pristine and UV-irradiated samples, and Table 4 provides a detailed summary of the calculated colour coordinates.

**Table 4:** Chromaticity coordinates of chitosan blends.

Coordinates	CHI-0		CHI-1		CHI-2	
	As prepared	UV- irradiated	As prepared	UV- irradiated	As prepared	UV- irradiated
X	0.3628	0.3424	0.3330	0.3973	0.3330	0.4006
Y	0.4084	0.4126	0.4006	0.3581	0.3973	0.3581

## Conclusion

In summary, this study investigated the effects of UV irradiation on the optical and electronic properties of Chitosan and two novel Chitosan derived polymers. All polymers exhibited characteristic amorphous Chitosan behavior. UV irradiation significantly increased the refractive index, particularly in the anomalous dispersion region, likely due to induced cross-linking and changes in electronic properties. While dielectric constants and some nonlinear optical parameters showed minor variations, UV exposure consistently decreased the band gap energy from 5.36 to 4.5 eV and altered the absorption spectra, suggesting modifications to the electronic structure. UV exposure increased the width of localized states (E_u_) to increase from 0.35 to 0.72 eV for CHI-1. Notably, enhanced nonlinear optical properties were observed in the 200–500 nm range after UV irradiation, demonstrating potential for applications in optical switching and limiting. Furthermore, all polymers exhibited white light emission, making them promising candidates for OLED applications. These findings demonstrate that UV irradiation can effectively tune the optical and electronic properties of chitosan-based materials, opening ways for their utilization in various optoelectronic devices.

## Data Availability

All data supporting the findings of this study are available within the paper.
